# Evaluation of Immigrant Tuberculosis Screening in Industrialized Countries

**DOI:** 10.3201/eid1809.120128

**Published:** 2012-09

**Authors:** Manish Pareek, Iacopo Baussano, Ibrahim Abubakar, Christopher Dye, Ajit Lalvani

**Affiliations:** Imperial College London, London, UK (M. Pareek, A. Lalvani);; University of Leicester, Leicester, UK (M. Pareek);; Univeristà degli Studi del Piemonte Orientale, Novara, Italy (I. Baussano);; Health Protection Agency, London (I. Abubakar);; University of East Anglia, Norwich, UK (I. Abubakar);; and World Health Organization, Geneva, Switzerland (C. Dye)

**Keywords:** tuberculosis, latent tuberculosis, tuberculosis and other mycobacteria, immigration, immigrants, screening, policies, practices, industrialized countries, epidemiology

## Abstract

In industrialized countries, tuberculosis (TB) cases are concentrated among immigrants and driven by reactivation of imported latent TB infection (LTBI). We examined mechanisms used to screen immigrants for TB and LTBI by sending an anonymous, 18-point questionnaire to 31 member countries of the Organisation for Economic Co-operation and Development. Twenty-nine (93.5%) of 31 responded; 25 (86.2%) screened immigrants for active TB. Fewer countries (16/29, 55.2%) screened for LTBI. Marked variations were observed in targeted populations for age (range <5 years of age to all age groups) and TB incidence in countries of origin of immigrants (>20 cases/100,000 population to >500 cases/100,000). LTBI screening was conducted in 11/16 countries by using the tuberculin skin test. Six countries used interferon-γ release assays, primarily to confirm positive tuberculin skin test results. Industrialized countries performed LTBI screening infrequently and policies varied widely. There is an urgent need to define the cost-effectiveness of LTBI screening strategies for immigrants.

## Abstract 2

Improvements are needed in current screening, which is insufficient and ineffective.

Tuberculosis (TB) in industrialized countries has reemerged as a public health concern after decreases in incidence during the 20th century. Over the past 30 years, although industrialized countries have shown country-specific quantitative changes (decrease, stabilization, or increase) in overall TB notifications, they share a similar underlying shift in TB epidemiology: decreasing incidence in the native population and an increasing incidence in foreign-born persons (*1*,*2*).

This disproportionate epidemiology is driven primarily by interaction of reactivating latent TB infection (LTBI) and high or increased immigration levels. This interaction is demonstrated by the small proportion of clustered cases among foreign-born persons, which is lower than that among native-born persons, in molecular epidemiology studies from diverse industrialized settings (*3*). This interaction is also demonstrated by TB acquired before immigration and high or increasing levels of immigration from countries with a high incidence of TB in sub-Saharan Africa, Asia, South America, and northern Africa to industrialized countries that have a low incidence of TB (*4*,*5*).

Surveillance data from several industrialized countries show that a high proportion of active TB cases in foreign-born persons occurs in the first 5 years after arrival (new entrants) (*6*,*7*). The high level of foreign-born persons with TB in industrialized countries potentially jeopardizes national TB control programs and has reopened the debate about how industrialized, immigrant-receiving countries should screen immigrants (*8*,*9*). Although industrialized countries have national policies on immigrant screening, little contemporary comparison (*10*) of critical elements of these policies has been made.

We conducted an international evaluation of screening practices for TB among immigrants in industrialized countries. We also compared critical elements of national guidance, including whether screening identified case of active TB or LTBI, which groups were targeted for screening, when screening was conducted, and which screening tools were used.

## Methods

### Ethics

No patient-specific data or personal identifiers were used. Our study was an analysis of routine data collected as part of service evaluation.

### Sampling Frame

All 31 industrialized (high-income) member states, as of 2010, of the Organisation for Economic Co-operation and Development (OECD) (Technical Appendix Table 1) were included in the study (*11*). These countries have an estimated population of 1.0 billion persons, of whom 109.5 million (10.95%; 95% CI 10.94%–10.95%) are immigrants (*12*). Six of the top 10 immigrant-receiving countries are industrialized OECD countries (*12*). In 2009, median TB incidence in these countries was 7.4 cases/100,000 population (interquartile range [IQR] 6.0–10.6 cases/100,000), and a median of 46.9% (IQR 30.1%–65.0%) cases were in foreign-born persons (*1*,*13*).

### Questionnaire

An 18-point questionnaire (Technical Appendix Table 2) based on a published evaluation of screening practices in the United Kingdom (*14*) was formulated to obtain information on immigrant screening practices in each industrialized country. Data were collected during May–December 2010 by abstracting published, publicly available, national immigrant TB screening guidelines or, more frequently, by contacting (by email) persons involved in local TB control programs and screening of immigrants for TB (usually the TB control program director). Replies were received electronically, and nonresponders were emailed 2 reminders. No person-specific data were collected on the questionnaire.

### Country-specific Data

Information for 2009 (or the most recent publically available data) on country-specific TB incidence was used. Countries with low and high TB incidence were classified as having <15 cases/100,000 and ≥15 cases/100,000, respectively, according to national TB reports (*15*–*20*). The proportion of cases among foreign-born persons were obtained from the World Health Organization global database (*21*), and net imigration rates were obtained from the OECD migration database (*22*).

### Statistical Analysis

Data were analyzed quantitatively, although certain open answers were categorized by investigators. Categorical responses were summarized by using proportions and 95% CIs, and comparisons were made by using the Fisher exact test. Continuous data were non-normally distributed and summarized as medians and IQRs and compared by using the Mann-Whitney U test. Analyses were performed by using STATA version 12.0 (StataCorp LP, College Station, TX, USA). A p value <0.05 was considered significant.

## Results

### Response Rate and Profile of Responding Countries

Data were obtained from 29 (93.5%) of 31 industrialized OECD countries (Technical Appendix Table 1). For these 29 countries in 2009, median TB incidence was 7.6 cases/100,000 (IQR 6.4–10.6), 46.8% (IQR 29.7%–64.6%) of cases were in foreign-born persons, and median net annual number of immigrants was 30,623 (IQR 12,322–77,206). There was no significant difference between responder and nonresponder countries for median TB incidence (p = 1.00), median proportion of cases in foreign-born (persons = 0.68), or median net number of immigrants (p = 0.36).

### Coverage and Extent of Immigrant Screening for Active TB

Twenty-five (86.2%) of 29 countries (95% CI 67.3%–96.0%) had a system for screening immigrants for active TB (Table 1; Technical Appendix Table 3); this system was compulsory in 19 (76.0%) of 25. Sixteen (64.0%) of 25 screened all legal immigrants and 4 (16.0%) of 25 screened selected legal immigrants. A higher number (24/25, 96.0%; p = 0.02) screened refugees/asylum seekers than all legal immigrants. Five (20.0%) of 25 countries restricted screening for active TB to refugees/asylum seekers. Countries that screened immigrants for active TB (either refugees/asylum seekers or legal immigrants) were less likely to have a high incidence of TB (≥15 cases/100,000) (50% vs. 95.7%; odds ratio 0.05, 95% CI 0.003–0.59, p = 0.018).

**Table 1:** Screening practices for detecting active tuberculosis in immigrants in 29 industrialized OECD countries*

Screen for active tuberculosis	No. countries positive/no. tested (%)
Yes	25/29 (86.2)
Compulsory	19/25 (76.0)
Timing of screening†	
Prearrival	9/25 (36.0)
At arrival	5/25 (20.0)
Postarrival	23/25 (92.0)
Type of immigrants screened	
All legal‡	16/25 (64.0)
Selected legal§	4/25 (16.0)
Refugees/asylum seekers¶	24/25 (96.0)
Selection criteria for immigrants screened	
Age	2/25 (8.0)
All ages	23/25 (92.0)
TB cases/100,000 population in country of origin	6/25 (24.0)
>15	1/6 (16.7)
>40	2/6 (33.3)
>50	2/6 (33.3)
>100	1/6 (16.7)
Region of origin#	19/25 (76.0)
All	5/19 (26.3)
All except EU, North America, Australia, and New Zealand	11/19 (57.9)
Other**	4/19 (21.1)
Screening tools used in children	
Clinical examination	6/25 (24.0)
Clinical examination and TST	8/25 (32.0)
Clinical examination and chest radiograph	0/25 (0.0)
Clinical examination, TST, and chest radiograph	2/25 (8.0)
TST	3/25 (12.0)
TST and chest radiograph	3/25 (12.0)
Chest radiograph	3/25 (12.0)
Screening tools used in adults	
Clinical examination	1/25 (4.0)
Clinical examination and TST	1/25 (4.0)
Clinical examination and chest radiograph	9/25 (36.0)
Clinical examination, TST, and chest radiograph	2/25 (8.0)
TST	1/25 (4.0)
TST and chest radiograph	2/25 (8.0)
Chest radiograph	9/25 (36.0)

*OECD, Organisation for Economic Co-operation and Development; TB, tuberculosis; EU, European Union; TST, tuberculin skin test. †Numbers do not add up to the total because some countries screened at >1 location. ‡Countries in which all legal immigrants (if they meet screening criteria) are screened (see Technical Appendix for more detailed categorization and definitions). §Countries in which only selected categories of legal immigrants (if they meet screening criteria) are screened (see Technical Appendix for more detailed categorization and definitions). ¶Countries in which refugees/asylum seekers are screened (see Technical Appendix for more detailed categorization and definitions). #Numbers do not add up to the total because the Czech Republic screened refugees/asylum seekers from all countries but legal immigrants only from selected countries. **Congo, Czech Republic, Israel, Kenya, Moldova, Mongolia, Nigeria, North Korea, Pakistan, Slovenia, South Korea, Tajikistan,Turkmenistan, Uzbekistan, and Vietnam.

### Timing of Screening for Active TB

Countries differed in when they screened for active TB (Table 1; Table 2); several countries tailored screening according to type of immigrant (refugee/asylum seeker vs. legal immigrant). Nine (36.0%) of 25 countries screened prearrival, 5 (20.0%) of 25 screened at arrival, and 23 (92.0%) of 25 screened postarrival. Of the 25 that screened postarrival, 8 (34.8%) of 23 also screened prearrival and postarrival but reserved screening postarrival mainly for asylum seekers/refugees.

**Table 2:** Selection criteria for screening immigrants for active tuberculosis in selected OECD countries*

Country	Timing of screening relative to arrival in host country				Active tuberculosis target groups and immigrants selected for screening			
	Prearrival	At arrival	Postarrival		Age	Threshold of TB incidence/100,000 population	Target countries/regions	Intended duration of residence that triggers screening, mo
Australia	Yes	No	Yes†		All	NA	All countries	>3–12‡
Austria	Yes	No	Yes†		All	NA	All countries except United States, Canada, and EU	N/U
Belgium	No	Yes§	Yes†		All	>50	NA	N/U
Canada	Yes	No	Yes†		All	>15¶	NA	N/U
Czech Republic	Yes	No	Yes		All		All (if refugee/asylum seekers); otherwise, Kenya, Congo, Moldova, Mongolia, Nigeria, Pakistan, Turkmenistan, Tajikistan, Uzbekistan, and Vietnam	>3 and applying for long-term stay
Finland	No	No	Yes		All	>50	NA	N/U
France	Yes¶	No	Yes		All	NA	All countries except those in EU and North America, and Japan, New Zealand. and Australia	>2
Germany	No	No	Yes		All	NA	All high-incidence countries	N/U
Greece	No	No	Yes		All	NA	All countries except those in EU	N/U
Iceland	No	No	Yes		All	NA	All countries except those in EU (except Bulgaria and Romania),and Australia, New Zealand, Switzerland, United States, and Canada	N/U
Ireland	No	No	Yes		All	>40	NA	>3
Israel	Yes	No	No		>6 mo	NA	Sub-Saharan Africa, Horn of Africa, and Southeast Asia	N/U
South Korea	No	No	Yes		All	NA	North Korea	N/U
Luxembourg	No	No	Yes		All	NA	All countries except those in EU	>3
The Netherlands	No	No	Yes**		All	NA	All countries except those in EU, and Australia, Canada, Iceland, Israel, Japan, Monaco, New Zealand, Norway, Surinam, Switzerland, and United States	>3
New Zealand	Yes	No	Yes†		≥11 y	NA	All countries except those in North America and EU	>6
New Zealand	Yes	No	Yes†		≥11 y	NA	All countries	>12
Norway	No	Yes	Yes†		All	NA	All countries except those in EU, and United States, Canada, Australia, Japan, and New Zealand	>3
Poland	No	No	Yes		All	NA	All countries except those in EU	N/U
Portugal	No	No	Yes		All	NA	All countries	N/U
Slovakia	No	Yes	Yes†		All	NA	All countries except those in EU	N/U
Slovenia	No	No	Yes		All	NA	Asia, Africa and eastern Europe	N/U
Sweden	No	No	Yes		All	>100	NA	N/U
Switzerland	No	Yes	No		All	NA	All countries	N/U
United Kingdom	Yes††	Yes	Yes		All	>40	NA	>6
United States	Yes‡‡	No	Yes†,§§		All‡‡,§§	NA	All countries‡‡,§§	Varies¶¶

*OECD, Organisation for Economic Co-operation and Development; TB, tuberculosis; NA, not applicable; EU, European Union; N/U, not known/unclear. †Postarrival screening for asylum seekers/refugees. ‡Immigrants from low-risk countries (American Samoa, Andorra, Antigua and Barbuda, Australia, Austria, Barbados, Belgium, Bermuda, Canada, Chile, Costa Rica, Cuba, Cyprus, Czech Republic, Denmark, Finland, France, Germany, Greece, Grenada, Iceland, Ireland, Israel, Italy, Jamaica, Jordan, Libyan Arab Jamahiriya, Liechtenstein, Luxembourg, Malta, Monaco, Netherlands, Netherlands Antilles, New Zealand, Norway, Oman, Puerto Rico, Saint Kitts and Nevis, Saint Lucia, San Marino, Slovakia, Slovenia, Sweden, Switzerland, Trinidad and Tobago, United Kingdom and Northern Ireland, United States, US Virgin Islands, Vatican City) require chest radiographs when applying for permanent stay; immigrants from medium-risk countries (Albania, Algeria, Anguilla, Argentina, Bahamas, Belize, Bosnia and Herzegovina, Brazil, Colombia, Cook Islands, Egypt, Fiji, French Polynesia, Guam, Hungary, Iran, Japan, Kuwait, Lebanon, Macedonia [the former Yugoslav Republic], Maldives, Mauritius, Mexico, Montenegro, New Caledonia, Panama, Poland, Portugal, Saint Vincent and the Grenadines, Samoa, Serbia, Seychelles, Singapore, Spain, Syria, Tahiti, Tonga, Tunisia, Turkey, United Arab Emirates, Uruguay, Venezuela, West Bank, and Gaza Strip.) are required to be screened for active TB if staying in Australia for >12 months or applying for permanent stay; immigrants from high-risk countries (all countries not listed above including China India, Indonesia, Malaysia, Pakistan, the Philippines, Russia, South Africa, South Korea, Thailand, and Vietnam) need to be screened for active TB if staying for >3 months or applying for permanent residence. §Asylum seekers screened at arrival and then twice during the years after arrival (1 year in Flanders and 2 years in Brussels and Wallonia). ¶Countries with a 3-year average incidence of smear-positive TB >15 cases/100,000 population. #Prearrival screening for Morocco, Tunisia, Turkey, and Poland; otherwise, postarrival in France. **Immigrants/asylum seekers/refugees >12 years of age (>25 years in some regions) in the Netherlands from countries with TB incidence >200 cases/100,000 are screened after the initial entry chest radiograph with half-year chest radiographs for 2 years postentry. ††Prearrival screening for selected countries: Ghana, Burkina Faso, Côte d’Ivoire, Togo, Niger, Kenya, Eritrea, Somalia, Democratic Republic of the Congo, Rwanda, Uganda (visitor applications), Tanzania, Pakistan, Bangladesh, Sudan, Thailand, Cambodia, and Laos. ‡‡Prearrival screening conducted by a panel of physicians outside the United States; in countries with a TB incidence ≥20 cases/100,000, immigrants >15 years of age are screened by medical examination and chest radiograph, immigrants 2–14 years of age are screened by medical examination and tuberculin skin test (TST)/interferon-γ release assay (IGRA) (and chest radiograph if TST/IGRA results were positive) and those <2 years of age who have been screened by medical examination, which if suggestive of active TB, is followed by TST/IGRA with or with or without chest radiograph. In countries with a TB incidence <20 cases/100,000, immigrants >15 years of age undergo chest radiograph and those <15 years of age undergo medical examination which, if suggestive of active TB, is followed by TST/IGRA with or without chest radiograph. §§Postarrival screening is either a repeat chest radiograph for an immigrant who was previously screened overseas or screening for active TB by medical examination or TST/IGRA with or without chest radiograph (if TST/IGRA results were positive) in immigrants >2 years of age who originated from all countries, are already resident in the United States, and are applying for adjustment of their visa status (undertaken by civil surgeons). ¶¶Any duration of stay or persons applying for long-term visa status.

### Demographic Characteristics of Immigrants Selected for Active TB Screening

Specific immigrants, in terms of age and country of origin, targeted for active TB screening are shown in Table 1 and Table 2. Twenty-three (92.0%) of 25 countries screened all age groups for active TB.

There was more variability in countries of origin that were targeted for screening. Six (24.0%) of 25 countries determined which immigrants should be screened on the basis of TB incidence in their country of origin. Incidence thresholds at which screening was initiated ranged from >15 cases/100,000 to >100 cases/100,000, although >40 cases/100,000 and >50 cases/100,000 were most commonly used.

Nineteen (76.0%) of 25 countries selected migrants for screening on the basis of the country from which they originated. Immigrants from countries with high incidence of TB were most commonly screened; 11 (57.9%) of 19 countries screened immigrants arriving from any region except for the European Union (EU), North America, Australia, and New Zealand.

### Methods of Screening New Immigrants for Active TB

Among countries that screened for active TB, screening methods are shown in Table 1 and Technical Appendix Table 4. In children and certain adults, such as pregnant women, for whom chest radiography is generally avoided, the most common methods of initial screening for active TB were clinical examination plus tuberculin skin test (TST) and clinical examination.

Adults and older children were assessed by using similar methods, although countries differed in the minimum age at which they used chest radiographs to screen for active TB (range birth to >18 years). Overall, screening by clinical examination plus chest radiograph and chest radiograph were the most frequent methods of screening adults for active TB.

### Coverage and Extent of Immigrant Screening for LTBI

The proportion of industrialized OECD countries that screened immigrants for LTBI is shown in Table 3. Sixteen (55.1%) of 29 countries screened immigrants for LTBI. Of these 16 countries, 11 (73.3%; compulsory in 7 [(63.6%] of 11), 2 (13.3%; compulsory in 0 [0.0%] of 2), and 15 (93.8%, compulsory in 8 [53.3%] of 15) screened for LTBI in all legal migrants, selected legal migrants, and asylum seekers/refugees, respectively (12 countries screened >1 immigrant group) (Technical Appendix Table 3). There was no difference in TB incidence, proportion of cases among foreign-born persons, and net migration when we compared countries that screened and did not screen for LTBI.

**Table 3:** Immigrant screening practices for latent tuberculosis infection in 16 industrialized OECD countries*

Screen for latent tuberculosis	No. positive countries/ no. tested (%)
Yes	16/29 (55.2)
Location of screening	
Prearrival	2/16 (12.5)
At arrival	2/16 (12.5)
Postarrival	16/16 (100.0)
Selection criteria based on age	
No age cutoff†	6/16 (37.5)
Age cutoff values for screening, y	10/16 (62.5)
<5	1/10 (10.0)
<15	2/10 (20.0)
<35	3/10 (30.0)
<40	1/10 (10.0)
Other	3/10 (30.0)
TB incidence in country of origin	5/16 (31.3)
TB cases/100,000 population at screening‡§	
>20	1/5 (20.0)
>40	2/5 (40.0)
>50	1/5 (20.0)
>100	1/5 (20.0)
>500	1/5 (20.0)
Specific country of origin§	13/16 (81.3)
Countries screened	
All	5/13 (38.5)
All except EU, North America, Australia, and New Zealand	5/13 (38.5)
Other¶	3/13 (23.1)
Screening tools used for LTBI#	
TST	11/16 (68.8)
TST and confirmatory IGRA	4/16 (25.0)
IGRA	3/16 (18.8)

*OECD, Organisation for Economic Co-operation and Development; TB, tuberculosis; EU, European Union; LTBI, latent TB infection; TST, tuberculin skin test; IGRA, interferon-γ release assay. †All ages screened. ‡Numbers do not add up to the total because certain countries (e.g., Ireland) screened at different incidence thresholds for children and adults. §Numbers do not add up to the total because certain countries base screening criteria on incidence threshold and country of origin. ¶Ethiopia, Ireland, and Slovenia. #Numbers do add up to the total because in some countries (e.g., United Kingdom and United States), >1 screening method is allowed or recommended.

### Timing of Screening for LTBI

As with screening for active TB, countries differed in when they screened for LTBI (Table 3), which depended on the status of the immigrant (Table 4). Two (12.5%) of 16 countries screened prearrival and 2 (12.5%) of 16 screened at arrival, although screening was reserved primarily for asylum seekers and refugees. LTBI screening was most frequently conducted postarrival in the host country (16/16, 100%).

**Table 4:** Location of and selection criteria for screening immigrants for latent tuberculosis in selected OECD countries*

Country	Timing of screening				Criteria by which migrants are selected for latent tuberculosis screening						Screening tools used
	Prearrival	At arrival	Postarrival		Type of immigrants		Age	TB incidence/ 100,000 population	Target regions	Intended duration of residence that triggers screening	
					Legal	Refugees/ asylum seekers					
Belgium	No	Yes	Yes		Yes (selected)	Yes	<5 y†	>50	NA	N/U	TST
Czech Republic	No	No	Yes		No	Yes	<15 y	NA	All	N/U	TST
France	No	No	Yes		Yes	Yes	<15 y		All countries except those in EU and North America, and Japan, New Zealand, and Australia	>2 mo	TST
Greece	No	No	Yes		Yes	Yes	All	NA	All	N/U	TST‡
Iceland	No	No	Yes		Yes	Yes	<35 y	NA	All countries except those in EU (except Bulgaria and Romania), and Australia, New Zealand, Switzerland, United States, and Canada	>1 y	TST
Ireland	No	No	Yes		Yes	Yes	<35 y	>40§; >500/sub-Saharan Africa§	>500/sub-Saharan Africa§	>3 mo	TST
Israel	Yes¶	No	Yes¶		Yes	No	>6 mo	NA	Ethiopia		TST
Luxembourg	No	No	Yes		Yes	Yes	All	NA	All	>3 mo	TST
The Netherlands	No	No	Yes		Yes	Yes	<12 y#	NA	All countries except those in EU, and Australia, Canada, Iceland, Israel, Japan, Monaco, New Zealand, Norway, Surinam, Switzerland, and United States	>3 mo	TST‡
Norway	No	Yes	Yes		Yes	Yes	<40 y	NA	All countries except those in EU, and United States, Canada, Australia, Japan, and New Zealand	>3 mo	TST and confirmatory IGRA
Portugal	No	No	Yes		No	Yes	All	NA	All	N/U	TST and confirmatory IGRA
Slovak Republic	No	No	Yes		Yes	Yes	All	NA	All countries except those in EU	N/U	TST and confirmatory IGRA
Slovenia	No	No	Yes		Yes (selected)	Yes	All	NA	Asia, Africa, and eastern Europe	N/U	IGRA
Sweden	No	No	Yes		No	Yes	All	>100	NA	N/U	TST
United Kingdom	No	No	Yes		Yes	Yes	<35 y	>40**	NA	>6 mo	TST and confirmatory IGRA or IGRA alone
United States	Yes††	No	Yes		Yes‡‡	Yes	2–14 y†† or >2 y§§	≥20†	All§§	N/U	TST or IGRA

*OECD, Organisation for Economic Co-operation and Development; TB, tuberculosis; NA, not applicable; N/U, not known/unclear; TST, tuberculin skin test; EU, European Union; IGRA, IGRA, interferon-γ release assay. †In general, children <5 years of age are screened for latent TB although pregnant women of any age can also be screened. ‡TST is mainly used although IGRA can be used optionally if diagnosis is unclear in confirming the TST result. §If <16 years of age; screening adults (16–35 years of age) from >500 cases/100,000 or from sub-Saharan Africa. ¶First step for TST for Jewish immigrants from Ethiopia is performed in Addis Ababa (i.e., prearrival) and the second step is performed postarrival in Israel. #Applies to persons who were not vaccinated with *Mycobacterium bovis* BCG; in some centers in the Netherlands, immigrants <25 years of age are screened for latent TB infection. **Previous threshold for United Kingdom was >40 cases/100,000 if <16 years of age and 500 cases/100,000 for sub-Saharan Africa if 16–35 years of age. ††In the US system, immigrants 2–14 years of age from countries with a TB incidence ≥20 cases/100,000 are screened with TST or IGRA prearrival in their country of origin. Some persons are therefore identified as having latent TB and are advised to seek medical attention on arrival in the United States. ‡‡In the US system, immigrants can be screened postarrival if their initial screening test results suggest that they have inactive TB. §§All immigrants >2 years of age who apply to have their visa status adjusted (status adjusters) are screened by TST or IGRA.

### Demographic Characteristics of Immigrants Selected for LTBI Screening

Details of which immigrant subgroups were targeted for LTBI screening are shown in Table 3 and Technical Appendix Table 4. In 16 countries that screened for LTBI, persons of a wide range of ages (birth to <40 years of age) were screened. Children and young adults were most commonly targeted for screening although 8 (50.0%) countries imposed no upper age limit for screening.

Selection of immigrants for screening of LTBI as determined by TB incidence in the country of origin or by specific countries of origin was conducted in 5 (31.3%) of 16 and 13 (81.3%) of 16 countries, respectively. Selection criteria in the United States and Ireland used TB incidence and country of origin (Table 4). The incidence threshold at which immigrants were screened for LTBI ranged from >20 cases/100,000 to >500 cases/100,000. Among 13 countries that screened for LTBI on the basis of specific countries of origin, 5 (38.5%) screened immigrants arriving from countries with a high incidence of TB outside the EU, North America, Australia, and New Zealand, and 5 screened immigrants from all countries.

### Methods of Screening Immigrants for LTBI

Screening methods used by 16 industrialized countries that screened immigrants for LTBI are shown in Table 4. The most commonly used screening protocol was TST (11/16, 68.8%). Six (37.5%) countries used the interferon-γ release assay (IGRA) when diagnosing LTBI, 3 countries used a stepwise TST plus confirmatory (IGRA) approach, 2 countries advocated single-step IGRA, and 1 country (United Kingdom) recommended TST and confirmatory IGRA (for persons ≤35 years of age) and single-step IGRA (for persons 16–35 years of age).

## Discussion

Increased attention is being given to TB among immigrants as a public health issue in industrialized countries (*8*), which underscores use of our data in determining how to best augment current TB control programs. Our international evaluation of immigrant screening policies among industrialized OECD countries indicated that although screening for active TB is frequently performed, LTBI screening is less common. Moreover, screening that is performed for active TB and LTBI varies among countries. Our results indicate that heterogeneity exists in screening location, selection criteria for which immigrant subgroups to screen, and screening methods used.

The primary objective of immigrant screening in industrialized countries appears to be to diagnose active TB, either before immigration or soon after arrival in the host country. Although diagnosing and treating infectious TB reduces transmission, data from numerous settings suggest that yields for active TB diagnosed at or around the time of migration are low: 0.35% in recent meta-analyses and lower in UK studies (*23*–*26*).

Although most countries in our study screened for active TB, screening was not universal, and countries differed in which immigrants they screened. Among countries that screened for active TB, asylum seekers and refugees were most commonly targeted for screening. Although these groups are at high risk for having TB infection and disease (because of poor social circumstances, inadequate housing, poor nutrition, and stress of migration), and yields and effects from screening are likely to be higher among them (*23*), they constitute only 2.1% of all immigrants to industrialized OECD countries and are likely to have a smaller role in TB epidemiology (*12*). However, a limitation of current national surveillance data is that it does not stratify TB cases among foreign-born persons by immigration status, which makes it impossible to know what proportion of TB cases arise from documented versus undocumented or illegal immigrants.

There was evidence of heterogeneity for specific countries of origin and TB incidence in countries of origin that were selected for active TB screening. Immigrants from countries with high incidence of TB were generally targeted. However, this targeting was partly modulated because free movement of citizens between EU member states indicated that citizens of certain EU nations with a high incidence of TB (particularly where countries based the decision to screen solely on country of origin) were not eligible to be targets for screening when migrating within the EU, although they would be targeted when migrating to countries (mainly non-EU) that based screening policy on TB incidence. In those industrialized countries that used TB incidence as the selection criteria, screening was performed at incidence thresholds from >15 cases/100,000 to >100 cases/100,000.

It is unclear why countries had such different policies although setting the threshold at a higher level may have increased the yield of screening. The specific immigrant subpopulations targeted (either by country of origin or TB incidence in country of origin) may reflect unique migration patterns to each OECD country (*5*), which may stem from colonial, historic, or linguistic links, financial resources, the current health care system, and infrastructure to deal with immigrants. A possible limitation of current screening protocols is that they may not target immigrants from regions with a high incidence of TB who arrive in industrialized, low-incidence settings, acquire citizenship, and then move to a country with a low incidence of TB (although this group might be small).

Similar variation was observed in screening tools used to diagnose active TB. Younger children were often screened by clinical examination with or without a TST, although making a diagnosis of active TB in children is often difficult when based on such limited evidence (*27*). Adults and older children were usually screened by chest radiograph, although the lower age limit at which chest radiographs were permissible varied from birth to 18 years of age. The wide range likely reflects reluctance to unnecessarily expose children to radiation and different, more adult-like patterns of pulmonary disease seen in older children (*28*).

The prevalence of active TB at entry is small and imported active disease that is detectable among immigrants arriving in their country of destination is not driving the increasing disease incidence seen in foreign-born persons in industrialized OECD countries (*24*). Moreover, because epidemiologic data suggest a high and increasing proportion of extrapulmonary TB in foreign-born persons, chest radiographs would play a limited role in diagnosis (*29*,*30*). This finding would limit screening systems that many industrialized countries currently use for adults. The UK Health Protection Agency has reviewed health in port regions and recommended urgent review of continued use of chest radiography as the initial diagnostic test for new entrants (currently underway) (*26*).

In contrast, TB epidemiology in OECD countries and molecular typing data indicated that reactivation of imported LTBI in the first few years after immigration is driving the increase in foreign-born persons with TB cases (*3*). Therefore, although screening for active TB is needed, without commensurate targeting of LTBI, screening is unlikely to control TB at a population level. We found that only half of industrialized countries screened immigrants for LTBI, and refugees/asylum seekers were most commonly targeted for screening. This finding indicates that screening legal immigrants for LTBI remains a low-priority TB control measure in industrialized countries, a potential gap that needs to be urgently addressed.

Among countries that screen for LTBI, there was heterogeneity in which immigrant subgroups were screened. For age, children and young adults were most commonly targeted because these groups have the highest risk for progression to active TB and are most likely to benefit from chemoprophylaxis. However, 47% of countries screened all age groups for LTBI, which suggests that in certain countries, older immigrants are given chemoprophylaxis, despite often-cited concerns about hepatotoxicity (*31*).

Similar variability was seen in which countries of origin of immigrants were targeted for screening. Among industrialized countries that selected immigrants on the basis of TB incidence in the country of origin, the TB incidence screening threshold ranged from >20 cases/100,000 to >500 cases/100,000. This wide variation likely reflects uncertainty about the optimal threshold at which to screen. Setting the incidence threshold too low would result in large numbers of immigrants needing to be screened. Thus, a low threshold would increase costs and likely overwhelm TB screening services, although many immigrants from lower-incidence countries often do not contribute to TB incidence in industrialized countries that have a low prevalence of LTBI (*32*). In contrast, if the incidence screening threshold is set too high, few immigrants would be screened, which means that a large proportion of the immigrant population that has LTBI, and subsequently converts to active TB, would be missed (*32*).

The most cost-effective policy option is likely to be to target at an intermediate incidence that balances, most cost-effectively, the numbers of immigrants being screened (and therefore associated costs) against prevalence of LTBI in the immigrant population (*33*). However, in many OECD countries, making cost-effective policy decisions about immigrant screening for LTBI is hampered by gaps in evidence in several areas, including which immigrant groups to screen (depending on TB incidence/country of origin), which screening methods to use, and which location is best for screening. This policy may partly explain variability in screening models adopted by OECD countries. These gaps could be appropriately addressed by obtaining prospective, multicenter data on prevalence of LTBI in immigrants and assessing performance of screening tools and outcomes of screening in different locations. This policy would enable investigators to calculate yields and relative cost-effectiveness of screening at different incidence thresholds (as was conducted recently in the United Kingdom [(*33*]), for different screening tools and in different locations, thereby enabling countries to formulate country-specific, evidence-based, immigrant screening policies.

LTBI screening methods also varied widely. The most commonly used screening method was TST. Although TST is widely used and inexpensive, it has poor specificity in *Mycobacterium bovis* BCG–vaccinated populations (e.g., immigrants arriving in industrialized countries), poor sensitivity in immunocompromised persons, and logistic drawbacks, including the need for a return visit and trained staff (*34*). Although data suggest that IGRAs have higher specificity and sensitivity than TST (*34*), their use was limited to 40% of industrialized countries as a confirmatory test for a positive TST result and increasingly as a single-step test to replace TST. This finding may reflect recent evidence that suggests that IGRAs are cost-effective and, if results are positive, can predict progression to active TB (*35*,*36*). However, the predictive power of IGRA for progression to active TB does not appear to be higher than that of TST (*37*). Empirical data are needed for relative performance of these tests in immigrant populations so that contemporary health economic analyses can conclude which screening modality is most cost-effective. Given the pivotal need for predictive power in improving cost-effectiveness of testing for LTBI (*33*), a more powerfully prognostic test would transform the cost-benefit equation for LTBI screening.

A major factor when considering the potential effect of screening for and treating LTBI is that suboptimal completion rates for chemoprophylactic regimens adversely affect efficacy of screening programs, thereby underscoring the need for adopting a patient-centered approach and new, faster-acting, drugs for LTBI (*38*). Given these potential drawbacks, an alternative approach, depending on patient preference and risk perception, could be to follow-up persons with LTBI for clinical signs over a defined period to rapidly identify and treat those with infections that become active. This approach is used in parts of the United Kingdom and the Netherlands (*39*).

Our study builds on previous research, which focused on fewer industrialized countries (*40*) and was conducted some years ago. However, it failed to capture recent changes in guidance (*40*), did not specifically focus on LTBI, and failed to identify the critical elements of immigrant screening programs, such as which immigrants were selected for screening (*10*,*40*).

Our study had several limitations. Information was gathered through a questionnaire with potential for recall/responder bias, although this limitation was minimized by clarifying ambiguous responses of responders or cross-referencing against national guidelines. In addition, our study only captured what screening is currently recommended, and this limitation presents an idealized situation of how screening should be conducted, which may be different from actual practice at the local level (*14*). Only a detailed assessment of national practice can determine the extent to which national guidance is followed.

TB in industrialized countries primarily occurs in foreign-born persons. Current immigrant screening policies in these countries focus primarily on identifying active TB. Although the contribution of active TB at the time of immigration is crucial, data from 2 large contemporary meta-analyses suggest that the prevalence of active disease in immigrants arriving from countries with a high incidence of TB remains relatively low (0.35%) (*23*,*24*), making cost-effectiveness and value of the current screening strategies uncertain. In contrast, epidemiologic data suggest that LTBI reactivation in immigrants plays a central role in determining national TB incidence. However, LTBI screening is paradoxically limited, and there is no consensus on which immigrants to screen and how to screen.

Addressing these issues is critical to effective TB control in industrialized countries, as is identification and treatment of persons with LTBI, and where control measures should be targeted while remaining vigilant about timely diagnosis and treatment for active disease. To address this problem effectively, robust evidence-based data are urgently needed to develop affordable, effective, and cost-effective policies on which immigrant subgroups to screen (*33*). Such policies will need to be developed in the context of nation-specific economic considerations, including resource availability and the funds policy makers are willing to spend to control the incidence of active TB.

Technical Appendix Definitions and other information regarding evaluation of immigrant tuberculosis screening in industrialized countries.
