# Supplementary material for: Evaluation of Immigrant Tuberculosis Screening in Industrialized Countries
Source: Emerg Infect Dis. 2012 Sep;18(9):1422–9. doi: 10.3201/eid1809.120128 (PMC3437731; doi:10.3201/eid1809.120128)
Supplement: Technical Appendix — Definitions and other information regarding evaluation of immigrant tuberculosis screening in industrialized countries. [file 12-0128-Techapp-s1.pdf]

Article DOI: http://dx.doi.org/10.3201/eid1809.120128

Evaluation of Immigrant Tuberculosis Screening in Industrialized Countries

Technical Appendix

Supplementary Definitions

We defined immigrant groups broadly in line with the definitions used by the International Organization for Migration (*1*).

Legal Immigrants

This group is akin to documented immigrants who encompass those persons who enter a country legally, remain in the country in accordance with their admission criteria, and are long-term migrants.

Legal immigrant: "A person who moves to a country other than that of his or her usual residence for a period of at least a year, so that the country of destination effectively becomes his or her new country of usual residence. From the perspective of the country of departure, the person will be a long-term emigrant and from that of the country of arrival, the person will be a long-term immigrant" (*1*).

We further subcategorized countries that screened legal immigrants into those that screened 1) all legal immigrants: this group included those countries that screened all legal immigrants if they met the specific country's screening criteria in terms of country of origin/tuberculosis (TB) incidence/age; and 2) selected legal immigrants: this group included those countries that screened specific categories of legal immigrants (irrespective of the category: examples included students and pre-employment screening of workers) if they met the specific country's screening criteria in terms of country of origin/TB incidence/age.

Refugees/Asylum Seekers

On the questionnaire, respondents self-indicated if and when refugees/asylum seekers were screened. Refugees and asylum seekers were defined in line with definitions used by the International Organization for Migration (*1*).

Refugee: “A person who, owing to a well-founded fear of persecution for reasons of race, religion, nationality, membership of a particular social group or political opinions, is outside the country of his nationality and is unable or, owing to such fear, is unwilling to avail himself of the protection of that country. (Art. 1(A) (2), Convention relating to the Status of Refugees, Art. 1A (2), 1951 as modified by the 1967 Protocol)” (*1*).

Asylum seeker: Persons seeking to be admitted into a country as refugees and awaiting decision on their application for refugee status under relevant international and national instruments. In case of a negative decision, they must leave the country and may be expelled, as may any alien in an irregular situation, unless permission to stay is provided on humanitarian or other related grounds” (*1*).

Technical Appendix Table 1. Industrialized OECD countries evaluated for immigrant tuberculosis*

Country	Responded	Data source
Australia	Yes	Published guidelines
Austria	Yes	Tuberculosis expert from country
Belgium	Yes	Tuberculosis expert from country
Canada	Yes	Published guidelines
Czech Republic	Yes	Tuberculosis expert from country
Denmark	No	None
Estonia	Yes	Tuberculosis expert from country
Finland	Yes	Tuberculosis expert from country
France	Yes	Published guidelines
Germany	Yes	Tuberculosis expert from country
Greece	Yes	Tuberculosis expert from country
Hungary	No	None
Iceland	Yes	Tuberculosis expert from country
Ireland	Yes	Tuberculosis expert from country
Israel	Yes	Tuberculosis expert from country
Italy	Yes	Tuberculosis expert from country
Japan	Yes	Tuberculosis expert from country
South Korea	Yes	Tuberculosis expert from country
Luxembourg	Yes	Tuberculosis expert from country
The Netherlands	Yes	Tuberculosis expert from country
New Zealand	Yes	Published guidelines
Norway	Yes	Tuberculosis expert from country
Poland	Yes	Tuberculosis expert from country
Portugal	Yes	Tuberculosis expert from country
Slovak Republic	Yes	Tuberculosis expert from country
Slovenia	Yes	Tuberculosis expert from country
Spain	Yes	Tuberculosis expert from country
Sweden	Yes	Tuberculosis expert from country
Switzerland	Yes	Tuberculosis expert from country
United Kingdom	Yes	Tuberculosis expert from country
United States	Yes	Published guidelines and tuberculosis expert from country

*OECD, Organisation for Economic Co-operation and Development.

Technical Appendix Table 2. Questionnaire used to evaluate immigrant tuberculosis in OECD countries*

Question no.	Question
1	Does (insert name of country here) screen immigrants for TB?
2	If so, do you screen immigrants for active TB, latent TB, or both?
3	In terms of screening for active TB, is this compulsory or voluntary?
4	Where is screening for active TB done: prearrival (in other words in the country of origin), at arrival, or postarrival?
5	Which countries are targeted for active TB screening?
6	Is there a specific TB incidence threshold which you consider to be high risk that requires screening? (e.g., if immigrant is from a country with a TB incidence >20 cases/100,000 population)?
7	Who is targeted for active TB screening: all immigrants, refugees, asylum seekers?
8	Which age groups are screened for active TB?
9	How is screening for active TB undertaken: chest radiograph alone or some other method? Do you screen immigrants for latent TB?
10	In terms of screening for latent TB, is this compulsory or voluntary?
11	Where is screening for latent TB performed: pre arrival (in the country of origin), at arrival, or postarrival?
12	Which countries are targeted for latent TB screening?
13	Is there a specific TB incidence threshold which you consider to be high-risk that requires screening for latent TB? (e.g., if immigrant is from a country with a TB incidence >20 cases/100,000 population)?
14	Who is targeted for latent TB screening: all immigrants, refugees, asylum seekers?
15	Which age groups are screened for latent TB?
16	How is screening for latent TB undertaken: e.g., tuberculin skin test or IGRA?
17	Is treatment for latent TB infection compulsory or voluntary?
18	If you have a copy of your official guidance, could who please send it to me?

*OECD, Organisation for Economic Co-operation and Development; TB, tuberculosis: IGRA, interferon-γ release assay.

Technical Appendix Table 3. Current screening practices for active and latent tuberculosis in industrialized OECD countries*

	Screening for active tuberculosis					Screening for latent tuberculosis infection				
			Groups screened for active TB					Groups screened		
			Legal immigrants		Refugees/asylum seekers			Legal immigrants		Refugees/asylum seekers
Country	Performed?	Compulsory?	All	Selected		Performed?	Compulsory?	All	Selected	
Australia	Yes	Yes	Yes	NA	Yes	No†	NA	NA	NA	NA
Austria	Yes	Yes‡	Yes	NA	Yes	No	NA	NA	NA	NA
Belgium	Yes	No§	No	Yes¶	Yes	Yes	No	No	Yes¶	Yes
Canada	Yes	Yes#	Yes	NA	Yes	No**	NA	NA	NA	NA
Czech Republic	Yes	Yes	Yes††	NA	Yes	Yes	Yes	No	No	Yes
Estonia	No	NA	NA	NA	NA	No	NA	NA	NA	NA
Finland	Yes	No	No	Yes‡‡	Yes	No	NA	NA	NA	NA
France	Yes	Yes	Yes	NA	Yes	Yes	Yes	Yes	NA	Yes
Germany	Yes	Yes	No	Yes§§	Yes	No	NA	NA	NA	NA
Greece	Yes	Yes	Yes	NA	Yes	Yes	Yes	Yes	NA	Yes
Iceland	Yes	Yes	Yes	NA	Yes	Yes	Yes	Yes	NA	Yes
Ireland	Yes	No	Yes	NA	Yes	Yes	No	Yes	NA	Yes
Israel	Yes	Yes	Yes	NA	No	Yes	No	Yes	NA	No
Italy	No	NA	NA	NA	NA	No	NA	NA	NA	NA
Japan	No	NA	NA	NA	NA	No	NA	NA	NA	NA
South Korea	Yes	Yes	No	No	Yes	No	NA	NA	NA	NA
Luxembourg	Yes	Yes	Yes	NA	Yes	Yes	Yes	Yes	NA	Yes
The Netherlands	Yes	Yes	Yes	NA	Yes	Yes	Yes	Yes	NA	Yes
New Zealand	Yes	Yes	Yes	NA	Yes	No	NA	NA	NA	NA
Norway	Yes	Yes	Yes	NA	Yes	Yes	Yes	Yes	NA	Yes
Poland	Yes	Yes	No	No	Yes	No	NA	NA	NA	NA
Portugal	Yes	No	No	No	Yes	Yes	No	No	No	Yes
Slovakia	Yes	Yes	Yes	NA	Yes	Yes	Yes	Yes	NA	Yes
Slovenia	Yes	No	No	Yes¶¶	Yes	Yes	No	No	Yes¶¶	Yes
Spain	No	NA	NA	NA	NA	No	NA	NA	NA	NA
Sweden	Yes	No	No	No	Yes	Yes	No	No	No	Yes
Switzerland	Yes	Yes	No	No	Yes	No	NA	NA	NA	NA
United Kingdom	Yes	Yes	Yes	NA	Yes	Yes	No	Yes	NA	Yes
United States	Yes	Yes	Yes	NA	Yes	Yes	No##	Yes	NA	Yes

*OECD, Organisation for Economic Co-operation and Development; TB, tuberculosis; NA, not applicable.
†No formal screening for latent TB but immigrants may have to accept postarrival health test results (if initial chest radiograph suggests inactive TB) in which further investigations (including repeat chest radiograph, sputum examination, and tuberculin skin test) are conducted, although this may differ from country to country.
‡For work permit and refugees/asylum seekers; otherwise, screening is voluntary.
§For students, a normal chest radiographic result may be needed for additional examinations.
¶Selected screening of immigrants through school/university but this is not systematic or formal.
#Although compulsory, postarrival screening cannot be enforced, although it may affect citizenship application.
**No formal screening for latent TB but postarrival medical surveillance for persons in whom initial chest radiograph suggests inactive TB.
††Immigrants from certain countries applying for a visa of ≥90 d or long-term residence permission have to provide documentary proof that they do not have TB.
‡‡Screening is comprehensive for adopted children but informal for all other immigrants.
§§Run at state level but not national; selected screening performed for Aussiedler (ethnic Germans mainly from the former USSR who resettled in Germany).
¶¶Selected screening performed for work permit (as part of preemployment screening).
##Not compulsory for recent immigrants but compulsory for status adjusters.

Technical Appendix Table 4. Initial mandatory screening methods for active tuberculosis in selected industrialized OECD countries*

Country	Children								Adults				
	Clinical examination	Clinical examination and TST	Clinical examination and chest radiograph	Clinical examination, TST, and chest radiograph	TST	TST and chest radiograph	Chest radiograph	Applicable age, y	Clinical examination	Clinical examination and TST	Clinical examination and chest radiograph	Clinical examination, TST, and chest radiograph	TS
Australia	Yes	No	No	No	No	No	No	<16	No	No	Yes	No	N
Austria	No	No	No	No	No	Yes	No	All	No	No	No	No	N
Belgium	No	No	No	No	Yes	No	No	<5	No	No	No	No	N
Canada	Yes	No	No	No	No	No	No	<11	No	No	No	No	N
Czech Republic	No	No	No	No	No	Yes	No	<15	No	No	No	No	N
Finland	No	No	No	No	No	Yes†	No	<7	No	No	No	No	N
France	No	Yes	No	No	No	No	No	<15‡	No	No	Yes	No	N
Germany	No	Yes†	No	No	No	No	No	<15	No	No	No	No	N
Greece	No	No	No	Yes	No	No	No	All	No	No	No	Yes	N
Iceland	No	Yes	No	No	No	No	No	<18§	No	No	Yes	No	N
Ireland	No	Yes	No	No	No	No	No	<16	No	No	Yes	No	N
Israel	Yes	No	No	No	No	No	No	<0.5	No	No	Yes	No	N
South Korea	No	No	No	No	No	No	Yes	All	No	No	No	No	N
Luxembourg	No	No	No	No	Yes	No	No	All	No	No	No	No	Ye
The Netherlands	No	Yes¶	No	No	No	No	No	<12#	No	No	No	No	N
New Zealand	No	No	No	No	No	No	No	<11	No	No	Yes	No	N
Norway	No	No	No	No	Yes	No	No	<15	No	No	No	No	N
Poland	Yes	No	No	No	No	No	No	<18	No	No	Yes	No	N
Portugal	No	No	No	No	No	No	Yes	All	No	No	No	No	N
Slovak Republic	No	No	No	Yes	No	No	No	All	No	No	No	Yes	N
Slovenia	No	No	No	No	No	No	Yes	All	No	No	No	No	N
Sweden	No	Yes	No	No	No	No	No	All	No	Yes	No	No	N
Switzerland	Yes††	No	No	No	No	No	No	All	Yes††	No	No	No	N
United Kingdom	No	Yes‡‡	No	No	No	No	No	<11	No	No	Yes	No	N
United States	Yes§§	Yes¶¶	No	No	No	No	No	<15	No	No	Yes##	No	N

Country	Children								Adults				
	Clinical examination	Clinical examination and TST	Clinical examination and chest radiograph	Clinical examination, TST, and chest radiograph	TST	TST and chest radiograph	Chest radiograph	Applicable age, y	Clinical examination	Clinical examination and TST	Clinical examination and chest radiograph	Clinical examination, TST, and chest radiograph	TS

*Although chest radiography may not be the initial screening tool (especially for children) if other screening tools (such as clinical examination or tuberculin skin test [TST]/interferon-γ release assay [IGRA]) suggest active tuberculosis (TB), chest radio
OECD, Organisation for Economic Co-operation and Development.
† IGRA can be used instead of TST.
‡Children <10 years of age have only a clinical examination; children 10–15 years of age have clinical examination and TST.
§Persons 18–35 years of age are screened as per methods used for children <18 years of age, i.e., clinical examination and TST.
¶In the Netherlands, screening with TST is performed only if the persons has not previously been vaccinated with *Mycobacterium bovis* BCG.
#In some centers in the Netherlands, persons not vaccinated with BCG and <25years of age are screened by TST.
**Immigrants >12 years of age (>25 years of age in some regions) from a country with TB incidence >200 cases/100,000 population are screened after the initial entry chest radiograph by six-month chest radiographs for 2 years (postentry).
††Questionnaire used to stratify risk.
‡‡IGRA may be used to confirm a positive TST result.
§§In the US system, immigrants <2 years of age from countries with a TB incidence ≥20 cases/100,000 and those <15 years of age from countries with a TB incidence <20 cases/100,000 are screened by only a clinical examination. If this result is pos
without chest radiograph id performed.
¶¶In the US system, immigrants 2–14 years of age from countries with a TB incidence ≥20 cases/100,000 are screened by clinical examination and TST (or IGRA). If any of these screening test results are positive/suggestive of active TB, a chest radi
##In the US system, immigrants >15 years of age from any country are screened by clinical examination and chest radiograph.

Reference

1. International Organization for Migration. Glossary on migration. Geneva: The Organization; 2004.
